# Family member reported symptom burden, predictors of caregiver burden and treatment effects in a goal-oriented community-based randomized controlled trial in the chronic phase of traumatic brain injury

**DOI:** 10.1186/s12883-024-03841-7

**Published:** 2024-09-10

**Authors:** Marianne Løvstad, Ida Maria Henriksen Borgen, Solveig Lægreid Hauger, Ingerid Kleffelgård, Cathrine Brunborg, Cecilie Røe, Helene Lundgaard Søberg, Marit Vindal Forslund

**Affiliations:** 1 Department of Research, Sunnaas Rehabilitation Hospital, Bjørnemyrveien 11, Bjørnemyr, 1453 Norway; 2 Department of Psychology, Faculty of Social Sciences, University of Oslo, Oslo, 0316 Norway; 3 Department of Physical Medicine and Rehabilitation, Oslo University Hospital, Oslo, 0424 Norway; 4 Oslo Centre for Biostatistics and Epidemiology, Oslo University Hospital, Oslo, 0424 Norway; 5 Department of Rehabilitation Science and Health Technology, Faculty of Health Sciences, Oslo Metropolitan University, Oslo, 0130 Norway; 6 Institute of Clinical Medicine, Faculty of Medicine, University of Oslo, Oslo, 0316 Norway

**Keywords:** Brain injury, Caregiver burden, Rehabilitation, Loneliness, Family

## Abstract

**Background:**

Family members are often affected by the long-term consequences of traumatic brain injury, but are rarely involved in rehabilitation programs in the chronic phase. We thus do not know what family members´ main concerns are in the chronic phase, what factors are associated with perceived caregiver burden, and whether family members´ health and functioning improves due to rehabilitation efforts received by the patients. This study explored family-members` functioning, predictors of caregiver burden and effect for family members of a goal-oriented intervention in the chronic phase of traumatic brain injury.

**Methods:**

Family members self-reported data measuring their caregiver burden, depression, general health, loneliness, and their evaluation of patient competency in everyday life, patient awareness levels, main problem areas (target outcomes) for the patient related to the brain injury, and demographic data were collected. Regression models were used to explore predictors of caregiver burden, and mixed models analysis was used to explore treatment effects.

**Results:**

In total, 73 family members were included, 39 in the intervention group and 34 in the control group. Moderate to high caregiver burden was reported by 40% of family members, and 16% experienced clinical levels of depression. Family member loneliness and their evaluation of the patient`s level of functional competency explained 57% of the variability in caregiver burden. There were no treatment-related changes in caregiver burden, family member depression or general health. At T2 there was however a significant reduction in how family members rated severity of target outcomes that the family members had nominated at baseline (-0.38, 95% CI, -0.75 to -0.02, *p* = 0.04), but not for the target outcomes the patients had nominated.

**Conclusions:**

A significant proportion of family members to patients in the chronic phase of TBI continue to experience challenging caregiver burden and emotional symptoms. Both family member-related and patient factors contribute to caregiver burden. Interventions targeting patient complaints do not automatically alleviate family members´ burden. It is important to address social support for family members early after injury, and there is a need for more interventions specifically targeting family members´ needs.

**Trial registration:**

The trial was registered at ClinicalTrials.gov, NCT03545594 on the 4th of June 2018.

## Introduction

Traumatic brain injury (TBI; 1) may cause a multitude of cognitive, somatic, emotional, behavioral and social problems that in turn may have a long-term negative impact upon participation and psychosocial functioning [2–4]. It is well-established that caring for persons with TBI may have negative effects also for family members´ emotional functioning [5–8], quality of life [9–12] and their experienced burden from caregiver responsibilities [13–15]. It has also been shown that family functioning as well as anxiety and depression in family members do not necessarily improve over the first 5 years [8], and that while some family members experience a stable or reduced level of burden, there is a sub-group that reports increased burden levels [13]. The level of burden and distress in family members is multi-factorially determined, with mixed findings regarding potential predictors. A recent scoping review [16] concluded that factors related both to the care recipient such as injury severity, functional disability and mental health, and factors related to the caregiver such as time used on care responsibilities, social support/loneliness, mental health and unmet treatment needs contribute. The vast majority of the 24 studies included in the review were performed within the first year after injury, and much less is known about predictors of caregiver burden in the chronic phase of injury.

The fact that the functional outcome of patients seems to play an important role in family member outcomes, could imply that family members would experience improvements in their own functioning when patients receive adequate rehabilitation for their symptoms. In clinical work, we typically assume that interventions that result in symptom relief for our patients will also more or less automatically be beneficial for family members. There is, however, little empirical evidence to support such an assumption, especially not in the chronic phase after injury. Despite the fact that TBI is increasingly acknowledged as a life-long condition with lasting treatment needs for many [2, 17], there is a lack of sound scientific knowledge about what may help patients and their families with their heterogeneous functional impairments. In the chronic phase, most patients with TBI are home dwelling and many live together with their family members. As many as one third of the patients live with unmet health care needs, particularly in the domains of cognition, emotional functioning, and work [18]. In this regard, it is interesting to note that influential systematic reviews of the entire field of cognitive rehabilitation in the later years [19, 20], have not directly covered the issue of family members´ role in rehabilitation. Our research group recently conducted a systematic review of community-based interventions addressing patients with longstanding symptoms after acquired brain injury [21], which included 49 studies on holistic, physical, or specific interventions. The studies were largely characterized by methodological weaknesses, rendering sound treatment guidelines impossible to establish. As a sub-analysis, we also looked at how many of the studies included in the systematic review addressed family members directly or involved family members in the intervention [22]. This revealed that only 20 (41%) studies involved a family member or significant other. The degree of family involvement varied from active involvement where the intervention was delivered to the dyad of participant and family member, to a passive role where family members were only included in assessment and psychoeducation. Likewise, a systematic review of family member interventions published in 2007 by Boschen et al. [23] found only 4 studies in the field of acquired brain injury, and the quality of these was rated low to moderate. Since then, studies involving families have assessed problem-solving training [24], solution-focused family therapy, psychoeducation, psychotherapy and stress-management [25], behavioral management [26], peer-mentoring [27], and family centered interventions [28] with promising, but diverging results. The literature thus shows that family members are often not systematically involved in brain injury rehabilitation research in a systematic way. One interesting and recent exception, however, was a study conducted by Winter and colleagues in the United States [29]. In a randomized controlled trial (RCT), 81 veterans with predominantly mild TBI´s and a key family member received an in-home rehabilitation program addressing target outcome areas that were identified by the participants. The intervention resulted in lower levels of depressive symptoms and burden of care in the family members [30].

In summary, since family members are often not included in rehabilitation research in later stages after TBI, we do not have a clear picture of what family members´ main concerns are in the chronic phase, what factors are associated with their perceived caregiver burden in this phase after injury, and whether family members´ health and functioning improves due to rehabilitation efforts.

Inspired by the study by Winter et al. [29], a pragmatic randomized controlled trial (RCT) exploring the effect of a goal-oriented and home-based intervention was recently conducted by our research group in a sample of Norwegian civilians experiencing persisting TBI-related problems at least two years after complicated mild, moderate and severe TBI. Participation of a family member was not an absolute inclusion criterion, but strongly encouraged. A detailed description of study design [31], feasibility [32], patient and family member reported problem areas [33], and goal attainment in the intervention group [34], has been published previously. Results from the RCT demonstrated positive outcomes for the patients related to the severity and management of their most troublesome TBI-symptoms, anxiety, and health-related quality of life [35].

## Aims

In the current paper, we have analyzed data from a subpopulation of the abovementioned RCT, i.e. participants with an included family member, in order to:

1. Describe family member-reported outcomes regarding:

A) Family members functioning (caregiver burden, depression, and general health).
B) Patients´ functional level (patient competency, severity of main TBI-related problems).

2) Assess predictors of caregiver burden in the chronic phase of TBI.
3) Explore the effect of the goal-oriented intervention for family member-reported outcomes regarding:

A) Family members´ functioning (caregiver burden, depressive symptoms, and general health).
B) Patients´ functional level (patient competency, severity of main TBI-related problems).

## Materials and methods

### Study participants, design, and setting

The data in the present study are derived from an RCT evaluating the effectiveness of a goal-oriented and individualized rehabilitation intervention in the chronic phase of TBI. This study was conducted as a collaboration between Oslo University Hospital (OUH) and Sunnaas Rehabilitation Hospital in South-Eastern Norway. Eligible participants were invited by letter, screened by phone, and, if eligible, invited to a baseline assessment at OUH. Patient eligibility criteria were age 18–72, with a TBI diagnosis in the acute phase and radiologically verified intracranial traumatic injury. The participants had to be ≥ 16 years old at the time of injury, at least two years post-injury, and living at home. Furthermore, they had to report ongoing TBI-related problems and/or reduced physical and mental health and/or difficulties with participation in their everyday life. Exclusion criteria were severe progressive neurologic or severe psychiatric disorders (including active substance abuse and violence), inability to provide informed consent, inability to participate in a goal-setting process, or insufficient fluency in Norwegian. Inclusion of a family member appointed by the patients was encouraged. Patients could nominate any family member or friend as they saw fit as their main family member. Exclusion criteria for family members were insufficient fluency in Norwegian or cognitive dysfunction to the degree that questionnaires could not be reliably filled out. In this paper we report patient data on those with a participating family member only. Study participants were included between June 2018 and December 2020.

All participants, including family members where applicable, went through a baseline assessment (T1) prior to allocation of the patient to either the control group or the intervention group by an independent researcher using a randomly generated number sequence. All participants were also assessed shortly after the end of the intervention period, at 4–5 months (T2), and again at 12 months post-inclusion (T3). Assessments were carried out at the outpatient clinic at OUH and/or over the phone with mailed questionnaires. For further details on the study design, please refer to the study protocol [31] and previous publications from the study [32–35].

### Interventions

The intervention was manualized and based on the study by Winter and colleagues [29]. The intervention group received a home-based intervention consisting of eight contacts over a four-month period. The intervention sessions were initially delivered as six home visits and two telephone calls. Due to the Covid-19 pandemic, some patients were followed up by phone only during the initial Norwegian lockdown in March–May 2020. A pragmatic solution was adapted to continue recruitment during the pandemic, and most participants included from May to December 2020 (*n* = 17) were offered one to two home visits (first, ±last), while six to seven meetings were conducted by videoconference or telephone.

Target problem areas were established during baseline assessment in both groups. In the first sessions, participants in the intervention group were asked whether they wished to start working on any of the targets problem areas or another TBI-related difficulty. When a problem area was chosen, SMART (Specific, Measurable, Achievable, Relevant, and Timed) goals were established [36]. with goal attainment scaling [37, 38], and [3] development of an Action Plan [29] consisting of strategies to achieve the goal(s). Included family members were actively invited to participate in all or as many intervention sessions as possible and were encouraged to participate in goal setting and action planning together with the participant and the therapist. Four therapists with TBI rehabilitation expertise delivered the intervention: a medical doctor, a psychologist, a physiotherapist, and a neuropsychologist. Each participant was followed up by the same therapist throughout the intervention. Participants in the control group received treatment as usual (i.e., their usual healthcare and rehabilitation services provided in the municipality), but no additional study-based treatment. For a more detailed description of intervention content and measures applied, see Borgen et al. [34].

### Measures

Below is a description of the subset of measures reported in this paper.

#### Demographics and injury related data

For both patients and family members, demographic data such as age, gender and educational level and family members´ work status was collected. Family member relationship type was dichotomized into spouse/domestic partner (spouse or domestic partner) vs. other (mother, father, sister, brother or other). Family member loneliness was assessed by the question “Do you ever feel lonely?” self-reported by the family member and was dichotomized into never/seldom vs. sometimes/often. Injury-related information was collected from the patient’s medical journal and included injury severity and time since injury. Injury severity was classified as mild, moderate or severe TBI, based on the lowest unsedated Glasgow Coma Scale [39] score during the first 24 h after injury.

#### Standardized questionnaires

Standardized questionnaires were administered to family members at T1, T2 and T3.

*Caregiver burden* in family members was measured with the Caregiver Burden Scale (CBS; 40), which is a generic 22-item scale developed to measure different dimensions of a caregiver’s subjective burden. The items are scored from 1 to 4 (1, not at all; 2, seldom; 3, sometimes; 4, often), where higher scores indicate higher burden. The scale provides a mean index total score, and mean index subscale scores for the five subscales: general strain (8 items), isolation (3 items), disappointment (5 items), emotional involvement (3 items), and environment (3 items) [41]. A mean index score was calculated by using the total sum score divided by the total number of items. A dichotomized variable was calculated with (1) low caregiver burden, and (2) moderate to high caregiver burden, in line with recommendations, where scores ≤1.99 are classified as low caregiver burden, and scores ≥2 are classified as moderate to severe caregiver burden [40, 41]. The continuous mean index score was used in the mixed models and regression analyses.

*Depressive symptoms* in family members were assessed with the Patients Health Questionnaire-9 (PHQ-9; 42) which has a possible range of 0–27 (best-worst). A dichotomized depression variable was calculated based on the recommended cut-off value ≥ 10, indicating depressive symptoms of clinical significance [42, 43]. The total score was used as a continuous variable in the mixed models and regression analyses.

*General health in family members* was assessed through self-report with the EuroQol 5 Dimensions (EQ-5D) visual analog scale (VAS) 0-100 (0 = worst health possible, 100 = best health possible) [44]. The index score was used as a continuous variable in the mixed models and regression analyses.

*Patient competency in daily activities* was assessed with the Patient Competency Rating Scale (PCRS) Patient Form (patient-reported) and Relative Form (family member-reported; 45, 46), with a score range from 0 to 150 (worst-best). The continuous total score was used in the mixed models and regression analyses. A PCRS discrepancy score variable was computed by subtracting patient scores from family member scores [47]. In line with recommended procedures [48], the PCRS discrepancy score was further computed into a dichotomized awareness variable with positive values (≥ 0) vs. negative values (< 0). Negative scores indicate that the patient evaluates the patient function to be better than what the family member does, i.e. suggesting reduced patient awareness of the TBI-related symptoms.

*Target Outcomes* (TO) or main TBI-related problem areas for patients were reported at baseline by both patients and family members through a semi-structured interview. The interview was conducted separately with the patient and the family member. The patients were asked “What is the main problem related to your TBI that you have experienced in the past month?”, whereas the family members were asked “What is the main problem that [patient name] has experienced in the past month which is related to the TBI?”. Their open-ended answers were transcribed with the respondents’ choice of words by the baseline assessor. This process was repeated twice to come up with the second and third most bothersome problem areas for the patient as reported by the patient and family member, respectively. Both patients and family members rated the level of severity in managing the patient-nominated TO´s on a five-point Likert scale from 0 (“not at all difficult”) to 4 (“extremely difficult”). In addition, the family members also rated the severity of the family member-nominated TO´s by replying to the following question: “What would you say is the most prominent difficulty or the main problem that XXX has had during the past month which is caused by the brain injury?” and “How difficult is this problem to handle?” on the same five-point Lickert Scale. This resulted in three severity scores per participant; (1) patient rating of patient-nominated TO, (2) family member rating of patient-nominated TO, and (3) family member rating of family member-nominated TO. The TO-severity measures were re-assessed at T2 and T3. The mean severity scores across the TO´s were calculated and used in the mixed models. The TO´s were categorized into four overarching domains (cognitive, physical, and emotional difficulties, and social function/participation) through a data-driven and consensus-based approach, as described in Borgen et al. [33].

### Data analysis and statistics

Descriptive statistics were used to establish baseline characteristics. As demographic and injury-related data were not normally distributed, group comparisons were conducted with Mann Whitney U-tests for continuous data, and chi-square analysis for categorical variables. Continuity correction was applied to the chi-square analysis for dichotomized variables (i.e., 2 by 2 tables). In the analyses related to aims 1 and 2 regarding family member-reported data and predictors of caregiver burden, data from the two treatment groups were collapsed. Uni- and multivariable linear regression analyses were performed to investigate factors associated with caregiver burden (CBS mean index score). Variables were included simultaneously in the multivariable models based on the existing knowledge-base and expert opinion. The following factors were included: family member age, relationship type, family member loneliness, family member depressive symptoms (PHQ-9 total score), family member self-reported general health status (EQ-5D VAS index score), family member-reported patient competency (PCRS total score), and awareness. We present the full multivariable model, without subsequent data driven elimination of variables. The results are presented as regression coefficients (B) with 95% confidence interval (CI), standardized regression coefficients (β), and explained variance (adjusted R^2^). Multicollinearity of the factors was explored using Pearson’s or Spearman’s correlation coefficient ≥ 0.7 as a cut-off. Normality of residuals were examined by Q-Q plot and histograms.

To explore treatment effects over time, separate linear mixed effect models were fitted to the continuous outcome variables caregiver burden (CBS mean index score), depression (PHQ-9 total score), general health (EQ-5D VAS index score), patient competency in daily activities (PCRS total score) and patient- and family member-nominated TO mean severity, with time, treatment, and time-by-treatment interaction as fixed effects. Based on the linear mixed effect models, mean values were estimated with 95% confidence intervals (CI) for all time points (T1, T2, and T3) for the family members in each treatment group (intervention/control). We also estimated mean within and between group difference in change from T1 to T2 and from T1 to T3. Statistical significance was set at 5%, and the *p*-values were two-tailed. IBM SPSS statistics version 28 (IBM Corp., Armonk, NY, USA) or STATA version 17 (StataCorp LP, College Station, TX, US) was used for the statistical analyses.

## Results

### Sample characteristics

In the total sample of 120 patients participating in the RCT, 78 (65%) appointed a family member they wished to include and who consented to participation. Five family members participated in the baseline assessment only, but did not take part in the treatment sessions, and did not provide data at T2 and T3. This leaves a total sample of 73 dyads of participants and family members. Of these, 39 were allocated to the intervention group, and 34 to the control group. The average study participant with TBI was male, in the 40’s and had finished high school. The family members were primarily spouses/domestic partners, or mothers. The family member group was mainly of working age, and the vast majority were working outside the home. Family members in the intervention and control groups were comparable with regards to type of relationship, age, education levels and work status (Table 1). The family members in the intervention group participated in a median of 4 (57%; IQR: 3–6) of the sessions. Only six (15.4%) family members participated in seven or all eight sessions.

**Table 1:** Study sample characteristics – family members and patients

	Intervention group (*n* = 39)	Control group (*n* = 34)	Total (*n* = 73)	Group comparison*
**Family members**				
Relationship type, n (%)				*p* = 0.31 (spouse/partner vs. remaining categories collapsed)
- Spouse/partner - Mother - Sibling/other	27 (69.2%) 8 (20.5%) 4 (10.3%)	28 (82.3%) 2 (5.9%) 4 (11.8%)	55 (75.3%) 10 (13.7%) 8 (11.0%)	
Age in years, median (IQR)	49.0 (25.0)	50.5 (25.0)	49.0 (25.0)	*p* = 0.34
Education level in years, n (%)				*p* = 0.21 (≤13 vs. >13 years)
- ≤10 - 11–13 - 14–16 - ≥17	4 (10.3%) 20 (51.3%) 10 (25.6%) 5 (12.8%)	1 (2.9%) 14 (41.2%) 18 (53.0%) 1 (2.9%)	5 (6.8%) 34 (46.6%) 28 (38.4%) 6 (8.2%)	
Work status, n (%)				*p* = 0.85 (working/student vs. remaining categories collapsed)
- Working - Student - Sick leave - Disability pension/ retired - Not employed	27 (69.2%) 1 (2.6%) 1 (2.6%) 7 (17.9%) 3 (7.7%)	25 (73.5%) 1 (3.0%) 0 (0.0%) 8 (23.5%) 0 (0.0%)	52 (71.2%) 2 (2.7%) 1 (1.4%) 15 (20.6%) 3 (4.1%)	
Family member loneliness, n (%)				0.29
- Never/seldom - Sometimes/often	22 (56.4%) 17 (43.6%)	14 (41.2%) 20 (58.8%)	36 (49.3%) 37 (50.7%)	
**Patients**				
Age in years, median (IQR)	35 (25)	50.5 (21,75)	47 (24.5)	*p* = 0.01
Number of men, n (%)	29 (74.4%)	26 (76.5%)	55 (75.3%)	*p* = 1.00
Education level in years, n (%)				*p* = 0.88 (≤13 vs. >13 years)
- ≤10 - 11–13 - 14–16 - ≥17	5 (12.8%) 22 (56.4%) 9 (23.1%) 3 (7.7%)	1 (2.9%) 24 (70.6%) 5 (14.7%) 4 (11.8%)	6 (8.2%) 46 (63.0%) 14 (19.2%) 17 (9.6%)	
TBI severity (*n* = 72), n (%)				*p* = 0. 30
- Mild complicated (GCS 13–15) - Moderate (GCS 9–12) - Severe (GCS 3–8) - Missing	9 (23.1%) 6 (15.4%) 21 (53.8%) 3 (7.7%)	13 (38.2%) 6 (17.7%) 13 (38.2%) 2 (5.9%)	22 (30.1%) 12 (16.4%) 34 (46.6%) 5 (6.9%)	
Time since injury in years, median (IQR)**	4.0 (3.3)	4.0 (5.0)	4.0 (3.8)	*p* = 0.97

IQR = Interquartile range (between 25th and 75th percentile).

*Group comparisons were conducted with independent samples Mann Whitney U-tests for continuous variables, and chi-square analysis for categorical variables (variables were dichotomized if any cell count was < 5)

**TBI severity assessed by lowest unsedated Glasgow Coma Scale Score (GCS) within 24 h after injury

### Aim 1 - family member-reported outcomes

Levels of caregiver burden, depression and general health, and family member-reported severity of main TBI-related problems (TO’s) and patient competency, are presented in Table 2 per group and time-point.

**Table 2:** Intervention effect on family member reported outcome measures from linear mixed models

			T1		T2	Within group change T2-T1	Between group change T2-T1		T3	Within group change T3-T1	Between group change T3-T1
		n	mean (95% CI)	n	mean (95% CI)	mean (95% CI) *p*-value	mean (95% CI) *p*-value	n	mean (95% CI)	mean (95% CI) *p*-value	mean (95% CI) *p*-value
Intervention effect on the family member outcomes	Caregiver Burden Scale (CBS). Mean index score										
	Control	34	1.82 (1.62, 2.02)	33	1.87 (1.67, 2.06)	0.05 (-0.09, 0.19), *p* = 0.50	-0.07 (-0.26, 0.12), *p* = 0.45	31	1.87 (1.67, 2.07)	0.05 (-0.09, -0.19), *p* = 0.48	-0.11 (-0.31, 0.08), *p* < 0.26
	Intervention	39	1.83 (1.65, 2.02)	37	1.81 (1.62,1.99)	-0.03 (-0.16, 0.10), *p* = 0.70		35	1.77 (1.58, 1.96)	-0.06 (-0.19, 0.07), *p* = 0.37	
	**Patient Health Questionnaire (PHQ-9)**,** total score**										
	Control	33	6.08 (4.53, 7.63)	32	5.90 (4.44, 7.36)	-0.18 (-1.47, 1.12), *p* < 0.79	0.29 (-1.48, 2.07), *p* = 0.74	31	5.89 (4.08, 7.70)	-0.19 (-2.14, 1.76), *p* = 0.85	0.31 (-2.38, 3.00), *p* = 0.82
	Intervention	38	4.26 (2.81, 5.71)	37	4.38 (3.00, 5.75)	0.12 (-1.09, 1.33), *p* < 0.85		34	4.38 (2.66, 6.11)	0.12 (-1.73, 1.98), *p* < 0.90	
	**EuroQol 5 Dimensions (EQ-5D) VAS**,** index score**										
	Control	31	72.19 (65.78, 78.59)	32	73.79 (67.44, 80.13)	1.60 (-4.64, 7.84), *p* < 0.62	-2.98 (-11.43, 5.46), *p* < 0.49	31	71.45 (65.04, 77.86)	-0.74 (-7.05, -5.57), *p* < 0.82	1.52 (-7.09, 10.13), *p* < 0.73
	Intervention	38	76.21 (70.35, 82.06)	37	74.82 (68.90, 80.74)	-1.38 (-7.08, 4.31), *p* = 0.63		34	76.99 (70.91, 83.07)	0.78 (-5.08, -6.65), *p* = 0.79	
**Intervention effect on the patient outcomes**	**Family member reported Patient Competency Rating Scale (PCRS)**,** total score**										
	Control	34	118.62 (113.40, 123.84)	33	116.89 (111.65, 122.13)	-1.73 (-4.83, 1.38), *p* < 0.28	2.39 (-1.89, 6.67), *p* = 0.27	31	118.40 (113.12, 123.68)	-0.22 (-3.40, 2.96), *p* = 0.89	0.96 (-3.41, 5.33), *p* = 0.67
	Intervention	39	119.21 (114.33, 124.08)	37	119.87 (114.95, 124.78)	0.66 (-2.28, 3.60), *p* < 0.66		35	119.95 (115.00, 124.90)	0.74 (-2.26, 3.75), *p* < 0.63	
	**Family member rating of Patient-nominated Target Outcomes**,** mean severity score**										
	Control	33	2.78 (2.52, 3.04)	33	2.18 (1.89, 2.47)	-0.60 (-0.88, -0.33), *p* < 0.001	-0.29 (-0.66, 0.09), *p* = 0.13	31	2.02 (1.65, 2.39)	-0.77 (-1.09, -0.44), *p* < 0.001	-0.01 (-0.45, 0.43), *p* < 0.97
	Intervention	39	2.53 (2.29, 2.77)	37	1.64 (1.36, 1.92)	-0.89 (-1.15, -0.64), *p* < 0.001		35	1.75 (1.41, 2.10)	-0.78 (-1.08, --0.47), *p* < 0.001	
	**Family member rating of Family member-nominated Target Outcomes**,** mean severity score**										
	Control	32	2.41 (2.16, 2.65)	31	2.12 (1.83, 2.41)	-0.29 (-0.55, -0.02), *p* = 0.03	-0.38 (-0.75, -0.02), *p* = 0.04	29	2.16 (1.79, 2.53)	-0.25 (-0.57, -0.08), *p* = 0.14	-0.42 (-0.87, 0.02), *p* = 0.06
	Intervention	36	2.33 (2.10, 2.56)	32	1.66 (1.39, 1.93)	-0.67 (-0.92, --0.42), *p* < 0.001		33	1.66 (1.31, 2.01)	-0.67 (-0.97, --0.37), *p* < 0.001	

Significant results (*p* < 0.05) are marked in bold

### Aim 1A: family members’ functioning (caregiver burden, depression, and general health)

At baseline, the group average mean index score on the CBS of 1.83 (SD 0.60) indicates that the sample had a relatively low caregiver burden. However, a large minority (*n* = 29; 39.7%) reported moderate to high caregiver burden at baseline, i.e. a mean index score of two or above. The emotional involvement subscale was the only one with a group average above the cut-off value; 2.02 (SD 0.81), indicating an experience of feeling annoyed at, or ashamed, and embarrassed of the person with TBI. The environment scale identifies potential problems in the physical environment that could affect the caregiver’s ability to take care of the patient, and had the lowest score with a mean of 1.39 (SD 0.57). The three remaining subscales all had mean scores of 1.8–1.94 (SD × s 0.71–0.76).

The group average depression scores at baseline of 5.1 (SD 4.4) were also well below established clinical cut-off values (≤10). However, 12 of the 73 family members reported PHQ-9 scores at 10 or above, indicating 16.4% with clinical levels of depressive symptoms.

The mean EQ-5D VAS score of 74.8 (SD 17.7) at baseline indicated that the family members at a group level rated their overall health as relatively good.

### Aim 1B: Patients´ functional level (patient competency and severity of main TBI-related problems)

At the group level, patient competency in everyday life (PCRS) total scores reported by patients (mean 117.9; SD 13.3) and family members (118.9; SD 14.5) did not differ significantly at baseline. However, awareness issues still seemed to affect a considerable minority of patients at an individual level, where as many as 34 (46.6%) showed a negative discrepancy in PCRS score when subtracted from family member scores, i.e. suggesting some level of reduced awareness or differences of appraisal of functioning in almost half the sample. Among the patients with awareness issues, the median discrepancy score was − 9 (IQR − 14.25 to -2.0).

All patients and family members nominated three main TBI-related problems (TOs), except five family members, who only nominated two. As noted in the Methods section, we have previously [34] published a classification of TO with 24 categories and four overarching domains: Cognitive, Physical/somatic, Emotional and Social & Participation. A detailed analysis at the category level could not be done in this study due to low case-numbers, but there was a significant (*p* < 0.001) difference in distribution at the domain level between patients and family members. Inspection of Table 3 shows that compared to the patients, family members reported fewer cognitive and physical, and more emotional, social and participation-related challenges. At baseline, the family members gave a mean severity rating on the family member-nominated TO´s of 2.4 (SD 0.70). Family members´ mean severity ratings of the patient-nominated TO´s was 2.6 (SD 0.76), which was significantly (*p* = 0.039) higher than the patient ratings of the same TO’s (mean: 2.4; SD 0.77).

**Table 3:** Frequency of Target Outcome domains reported by patients and family members at baseline

Target Outcome domain	Patient TO	Family member TO
Cognitive difficulties	75 (34%)	61 (28%)
Physical difficulties	90 (41%)	73 (33%)
Emotional difficulties	32 (15%)	45 (21%)
Social function and participation	22 (10%)	35 (16%)
Missing	0	5 (2%)
Total number of TOs, n (%)	219 (100%)	214 (100%)

TO = Target Outcome

### Aim 2: predictors of caregiver burden in the chronic phase of TBI

Results from multivariable linear regression examining factors associated with the CBS mean index score at baseline are shown in Table 4. The model shows that family member loneliness and family member reported patient competency (PCRS total score) were significantly associated with caregiver burden. The level of family member self-reported depressive symptoms approached significance. The family members’ age, relationship type and general health status, and patient awareness, were not associated with caregiver burden. The model in total explained 56.8% of the variance (adjusted R^2^) of caregiver burden in the sample.

**Table 4:** Multivariable linear regression model of factors associated with the Caregiver Burden Scale at baseline (*n* = 73)

Independent variables	Coefficients		95% CI	*P*-value
	B	β		
Family member age	0.005	0.117	-0.002, 0.012	0.173
Family member relationship type	-0.171	-0.122	-0.414, 0.072	0.164
Family member loneliness	0.494	0.410	0.270, 0.719	**< 0.001**
Family member depressive symptoms	0.024	0.174	-0.002, 0.050	0.073
Family member self-reported general health status	0.004	0.116	-0.002, 0.010	0.220
Family member reported patient competency	-0.020	-0.478	-0.028, -0.012	**< 0.001**
Awareness	-0.029	-0.024	-0.231, 0.172	0.771
**Adjusted R** ^**2**^	0.568			

B = Unstandardized. β = Standardized. Significant *p*-values are marked in bold

### Aim 3: family member reported effects of the intervention

See Table 2 for results from the linear mixed models analyses.

### Aim 3A: family member functioning

The model did not reveal significant between or within group differences from T1-T2 or from T1-T3 regarding family member reported caregiver burden, depression, or general health, indicating that the intervention did not affect the family members’ health directly.

### Aim 3B: Patients´ functional level

**Fig. 1:**
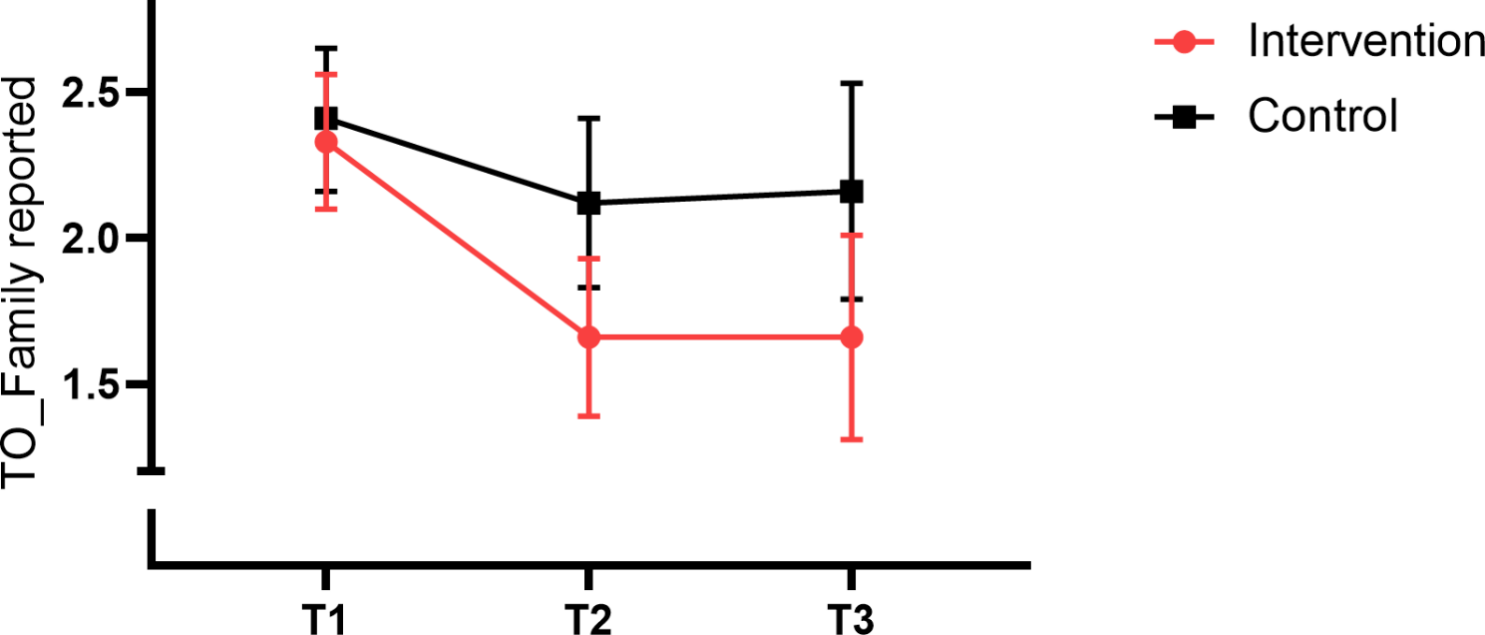
Severity of family member-reported Target Outcomes (TO) by group and time

There were also no between– or within-group changes in family member reported functional competency in the patients (PCRS). Regarding changes in how family members rated the severity of the TOs that were nominated by the patients, no significant between-group differences were observed, although there were significantly reduced severity ratings in both the groups from T1-T2 and from T1-T3. However, and interestingly, family member ratings of the TOs they had nominated themselves, showed that there was a specific change in favor of the intervention group from T1-T2 (*p* = 0.04). This effect was not maintained at T3, although the result was near-significant with *p* = 0.06. At the within-group level, a significant decrease in severity rating of the family member-nominated TO was seen in both groups from T1-T2, but only in the intervention group from T1 to T3. Figure 1 illustrates the significant group effect for severity rating of family member nominated TO´s.

## Discussion

In summary, the current study explored the characteristics of a sample of family members of persons with TBI in the chronic phase, predictors of caregiver burden and the effect of a goal-oriented intervention for family member reported outcomes.

The family members included by the patients were predominantly spouses and cohabiting partners, which is as expected, given a patient mean age in the mid 40´s. Two thirds of the family members were in paid work, which is representative for the Norwegian population at large [49]. The group means indicate that the family members experience low levels of care burden and normal levels of depressive symptoms, and that a considerable part of their caregiver burden is associated with emotional issues, as the emotional involvement subscale from the CBS had a group average corresponding to a moderate caregiver burden. Overall caregiver burden scores were comparable to those reported in the US study our intervention was modelled from [30] but slightly lower than earlier studies from a national Norwegian cohort of severe TBI at one and two years post injury [13, 50]. In this previous cohort, the environmental subscale was similarly the subscale with lowest burden at two years after injury. In contrast to our findings, the general strain, disappointment, and isolation subscales were all higher than the emotional involvement subscale in the previous sample. Lower mean subscale index scores could be explained by mixed severity of TBI in the present sample, and that longer time has passed since injury. The self-reported general health was 72 at baseline and 76 at T3, which is marginally below Norwegian population norms that have indicated a mean of 78 [51]. However, the group average data conceal a large sub-group of family members that experience a significant care burden and clinically significant emotional problems.

When it comes to how family members rated the main TBI-related problem areas´ severity (TOs), an interesting finding was that the TO´s reported by patients were rated as more severe by family members than by the patients themselves. This aligns well with the fact that discrepancy scores on the PCRS indicated that almost half of the patients rated their functional level as higher than the family members did. It is well known that many persons with TBI underestimate their level of impairment compared to the perception of the significant others´, i.e. that the individuals have awareness issues. Our finding that almost half of the sample experienced some level of reduced awareness, is supported by a previous study showing that 54% in a TBI sample of mixed severity displayed awareness issues 6 months after injury when this was indexed by discrepancy scores between patient and caregiver reports [52]. Of note, this study used a different measure than this study did and is not directly comparable. Also, it is important to note that providing an objective measure of awareness is difficult, if not impossible. Measuring awareness as a discrepancy between patients and family members is common, but problematic in that it implies that family member scores represent a gold standard of actual functioning. This is not always the case, as factors related to the family members such as overprotection, denial, or depression may bias their response style.

Another interesting finding was the functional domains covered by the main problem areas reported. While ¾ of the patient-reported TO´s were related to cognitive and physical problems, only 2/3 of the family reported problems were within these two domains. On the other hand, family members tended to report emotional, social and participation related problems as more predominant. This again confirms that the perspectives of those with the injuries and their close ones differ. This is probably not only due to lack of awareness, but also the fact that cognitive and physical problems such as fatigue, are not directly visible to others except through the behavioral consequences of the symptoms. Thus, family members tend to detect problems that affect the way patients interact with their surroundings through emotional and social behavior. This highlights the importance of including family members, as their perspectives on symptom burden and rehabilitation needs may differ from that of the patient.

The evaluation of factors associated with caregiver burden in the chronic phase produced interesting findings. Demographic factors such as age and type of relationship to the patient, general health and awareness issues in the patient did not predict caregiver burden levels, whereas family member loneliness and their perception of the patient’s functional status had high predictive value, and depression approached significance. Previous Norwegian studies have shown that both experienced unmet treatment needs [53] in families and caregiver burden [13] may increase from the first to the second year after injury. Manskow and colleagues [50] found that one year after severe TBI, caregiver burden was associated with social network, loneliness and disability levels in the patients, and also that experienced loneliness predicted increased caregiver burden over time [13]. We did not include a specific measure of social support, but interpret the findings related to loneliness as a proxy of social support. Overall, our findings align well with the existing literature in showing that both patient- and family member factors contribute to caregiver burden [16], and that a combination of loneliness/lack of social support and high disability levels in the patients is a particularly negative combination [13, 50, 54]. The fact that the model explained as much as 57% of the variance in caregiver burden was surprising. The finding is clinically important and points towards the importance of addressing not only the patients´ symptom burden, but also family members´ emotional and social situation when identifying families in need of rehabilitation services in the chronic phase. Loneliness was almost as strongly related to caregiver burden as patients´ functional levels, which underscores the importance of addressing the social situation of families in the early stages after injury, in order to prevent long-term social isolation.

Regarding the effects of the goal-oriented intervention, the lack of treatment related changes in caregiver burden, depression and general health confirms that we should not expect that family members´ perception of their own situation improves automatically when patients receive rehabilitation. Also, the finding is not overly surprising, as the intervention was primarily designed to address the patients’ self-determined main problems, with the support of family members. Another likely explanation could be that the sample size is too low to identify intervention effects on family member outcomes.

This study does, however, support the need for interventions that not only include family members as support to alleviate patients´ symptoms, but that they need help that directly addresses their own challenges. As noted in the Introduction, little research has been done in this area, and more family-centered intervention studies in the chronic phase are needed. The fact that we found intervention-related changes in the problem areas that the family members had nominated themselves, but not in those reported by the patients, supports this interpretation. The results from our RCT [35] showed that the intervention resulted in lower severity of patient-reported main TBI-related problem areas. Thus, both members of the patient-family member dyad seems to detect change more easily in the areas they themselves experience as most problematic, pointing to the need to take idiosyncratic factors into account when measuring treatment outcomes.

### Limitations

The family member group in this study is not necessarily representative of family members of persons with TBI in the chronic phase at large, as the group is derived from an RCT where the persons with TBI all identified TBI-related symptoms they were motivated to receive treatment for. On the other hand, the sample is for the same reason likely quite representative of patients that present themselves to rehabilitations centers with a need for symptom alleviation. The present study included a subpopulation of 60.8% of the total RCT-sample, and the sample size is a limitation. The effect sizes in Table 2 are relatively small, indicating that a larger sample would be needed to detect real, but small effects. The randomization was not stratified on participation of family member, resulting in a slight group size difference between the intervention and control group, and a difference between the groups regarding patients´ age. Given the finding that loneliness seems to play such an important role, it is a limitation that loneliness was covered with one question only, and that no specific measure of social support was included. Also, this intervention, like most rehabilitation trials, was a complex intervention, where the active treatment ingredients are probably complex and diverse between participants, complicating causal interpretations. Also, change might have occurred in both groups due to the additional attention, visits and assessments participating implied. On the other hand, positive group differences in favor of the intervention group does imply that efficacy of the intervention as such.

## Conclusions

The current study explored family member functioning in the chronic phase of TBI and confirms that a significant minority continues to experience significant caregiver burden and emotional symptoms, despite being in paid work at levels comparable to the general workforce. Family member loneliness and emotional functioning, along with functional impairments in the patients is particularly relevant for long-term caregiver burden, pointing towards the importance both of active rehabilitation efforts and a focus on family members psychosocial situation in the chronic phase of injury. The intervention findings indicate that family members will be most prone to detect change in the patients when the intervention addresses issues that the family members find important. The study also indicates a need for more intervention studies addressing the specific situation of family members, as rehabilitation efforts aimed at helping the patients does not necessarily influence family members well-being.

## Data Availability

The datasets analyzed during the current study are not publicly available due to ethical and legal regulations regarding protection of personal data.

## Abbreviations

CBS: Caregiver Burden Scale
GCS: Glasgow Coma Scale
OUH: Oslo University Hospital
PCRS: Patient Comptency rating Scale
PHQ-9: Patients Health Questionnaire
RCT: Randomized controlled trial
SRH: Sunnaas Rehabilitation Hospital
TO: Target Outcomes
TBI: Traumatic Brain Injury
